# What do we know about how to do audit and feedback? Pitfalls in applying evidence from a systematic review

**DOI:** 10.1186/1472-6963-5-50

**Published:** 2005-07-13

**Authors:** R Foy, MP Eccles, G Jamtvedt, J Young, JM Grimshaw, R Baker

**Affiliations:** 1 Centre for Health Services Research, University of Newcastle upon Tyne, Newcastle upon Tyne, United Kingdom; 2 Department for Health Technology Assessment, Reviews and Dissemination, Norwegian Health Services Research Centre, Oslo Norway; 3 Surgical Outcomes Research Centre, Central Sydney Area Health Service and University of Sydney, Royal Prince Alfred Hospital, Camperdown Australia; 4 Clinical Epidemiology Programme, Ottawa Health Research Institute, Ottawa Canada; 5 Department of Health Sciences, University of Leicester Leicester, UK

## Abstract

**Background:**

Improving the quality of health care requires a range of evidence-based activities. Audit and feedback is commonly used as a quality improvement tool in the UK National Health Service [NHS]. We set out to assess whether current guidance and systematic review evidence can sufficiently inform practical decisions about how to use audit and feedback to improve quality of care.

**Methods:**

We selected an important chronic disease encountered in primary care: diabetes mellitus. We identified recommendations from National Institute for Clinical Excellence (NICE) guidance on conducting audit and generated questions which would be relevant to any attempt to operationalise audit and feedback in a healthcare service setting. We explored the extent to which a systematic review of audit and feedback could provide practical guidance about whether audit and feedback should be used to improve quality of diabetes care and, if so, how audit and feedback could be optimised.

**Results:**

National guidance suggests the importance of securing the right organisational conditions and processes. Review evidence suggests that audit and feedback can be effective in changing healthcare professional practice. However, the available evidence says relatively little about the detail of how to use audit and feedback most efficiently.

**Conclusion:**

Audit and feedback will continue to be an unreliable approach to quality improvement until we learn how and when it works best. Conceptualising audit and feedback within a theoretical framework offers a way forward.

## Background

A range of strategies exist to promote the uptake of clinical research findings into the routine care of patients. They seek to change the behaviour of healthcare professionals and thereby improve the quality of patient care (Table 1). For each of these strategies a number of trials of their effectiveness have been drawn together within systematic reviews.[1,2] By examining interventions in a range of settings and circumstances such reviews aim to produce generalisable messages about the effectiveness or otherwise of these interventions.

**Table 1:** Examples of interventions to promote professional behaviour change.

*Educational outreach visits*	A personal visit by a trained person to a health care provider in his or her own setting
*Reminders (manual or computerised]*	Prompts performance of a patient specific clinical action
*Interactive educational meetings*	Participation of health care providers in workshops that include discussion or practice
*Audit and feedback*	Any summary of clinical performance over a specified period of time
*Local opinion leaders*	Health professionals nominated by their colleagues as being educationally influential
*Local consensus process*	Inclusion of professionals in discussions to agreed the approach to managing a clinical problem that they have selected as important
*Patient mediated interventions*	Specific information sought from or given to patients
*Educational materials*	Distribution of recommendations for clinical care (such as clinical practice guidelines, audio-visual materials, electronic publications).
*Didactic educational meetings*	Lectures with minimal participant interaction
*Financial incentives*	payments directly rewarding health care providers for specified behaviours
*Multifaceted interventions*	A combination of two or more interventions

All healthcare systems are concerned with improving the quality of care that they deliver as demonstrated by their establishment of structures (such as the UK NHS National Institute for Clinical Excellence (NICE], the Australian National Institute for Clinical Studies) and high profile reports.[3] Across countries clinical audit (hereafter referred to as audit and feedback) is commonly used to both monitor and improve quality of care. [4,5]

The strategies in Table 1 vary considerably in their resource requirements and cost effectiveness and any healthcare system will have finite resources to commit to quality improvement activities. Therefore to make the best use of health service resources, interventions to change professional behaviour should be evidence-based, selected on the basis of their known effectiveness and efficiency, and should be directed towards important clinical conditions.

While rising prevalence and changing patterns of service delivery diabetes mellitus increasingly contributes to the primary care workload [6] and there is evidence of fragmented and variable provision of care.[7] This paper explores the utility of current systematic review evidence to support healthcare system decisions about how to provide evidence based audit and feedback to improve the quality of care by considering it in the context of a common chronic condition and setting – diabetes mellitus in primary care. We aimed to find out whether we could operationalise audit and feedback from existing review data.

## Methods

### Topic selection

The UK NHS has produced a framework and set of measurable criteria by which to judge the quality of care for patients with diabetes mellitus. The National Service Framework (NSF) for Diabetes was launched in 2002.[8] It suggests performance targets for primary care organisations, responsible for the commissioning and provision of health care for defined populations. Some of these targets have been incorporated into the revised contract for UK primary care doctors (GPs) reflecting disease monitoring (e.g. HbA1c measurement) or secondary prevention (e.g. proportion of patients with HbA1c under 7.5%).[9] Therefore, diabetes represents an appropriate condition with which to explore the utility of audit: it is a common condition with important consequences, effective interventions are available, measurable outcomes have been defined, and there is potential for improvement in the quality of care.

### How best to conduct audit and feedback?

We informed the study with two definitions of audit and feedback (Table 2). The systematic review [5] offers a narrower definition than the National Institute for Clinical Excellence, *Principles for Best Practice in Clinical Audit *[4] which offers a broader definition and stresses the importance of integrating audit within an overall quality improvement framework. The latter sets out practical considerations for five stages of the audit and feedback process: preparing for audit, selecting criteria, measuring performance, making improvements, and sustaining improvement (Table 3). Much emphasis is given to creating the right organisational structures and culture for success, as well as taking account of local knowledge, experience and skills. Both are relevant to quality improvement at an organisational as well as individual level. However, neither describes in detail the manner in which audit and feedback should be conducted.

**Table 2:** Definitions of audit

**Definition of audit endorsed by the National Institute for Clinical Excellence **[4]

A quality improvement process that seeks to improve patient care and outcomes through systematic review of care against explicit criteria and the implementation of change. Aspects of the structure, processes, and outcomes of care are selected and systematically evaluated against explicit criteria. Where indicated, changes are implemented at an individual, team, or service level and further monitoring is used to confirm improvement in healthcare delivery.

**Definition of audit used by the Cochrane systematic review **[5]

The provision of any summary of clinical performance over a specified period of time. The summary may include data on processes of care (e.g. number of diagnostic tests ordered), clinical endpoints (e.g. blood pressure readings), and clinical practice recommendations (proportion of patients managed in line with a recommendation).

**Table 3:** Guiding principles for clinical audit.[4]

*Stage*	*Recommendations*	*Addressed within Cochrane Review?*
**Preparing for audit**	Securing stake-holder interest and involvement (e.g. professionals, patients or carers)	No
	Selection of appropriate topic, according to whether:	
	• Topic concerned is of high cost, volume, or risk to staff or users	No
	• Evidence of a serious quality problem	Yes: effects greater if low baseline
	• Good evidence available to inform quality standards	No
	• Amenability of problem to change	No
	• Potential for involvement in a national audit project	No
	• Topic is pertinent to national policy initiatives	No
	• Topic is a priority for the organisation	No
	Clear definition of purpose of audit, e.g. to improve or ensure the quality of care	No
	Provision of necessary support structures, i.e.	
	• Structured audit programme (committee structure, feedback mechanisms, and regular audit meetings)	No
	• Sufficient funding (audit staff, time of clinical staff, data collection, feedback)	No
	Identification of skills and people needed to carry out the audit	No
**Selecting criteria**	Definition of criteria (structure, process and outcome)	No
	Validity and potential to lead to improvements in care	
	• Evidence based	No
	• Related to important aspects of care	No
	• Measurable	Yes (implicitly)
**Measuring level of performance**	Planning data collection	
	• Definition of user group (and exceptions)	Can't tell
	• Definition of healthcare professionals involved	Yes (implicitly)
	• Definition of time period over which criteria apply	Yes (implicitly)
**Making improvements**	Identification of barriers to change	No
	Implementing change	
	• Establishing the right environment (at individual, team and organisational levels)	No
	• Considering external relationships (e.g. with patients or other agencies)	No
	• Use of other supporting interventions (e.g. educational outreach, reminders) and / or multifaceted interventions	Yes: not supported by evidence
**Sustaining improvement**	Monitoring and evaluating changes, e.g. continuing audit cycle, use of performance indicators	No
	• Appropriate organisational development (e.g. cultural change, adequate training)	No
	• Use of existing strategic, organisational or clinical frameworks	No
	• Leadership	No

We explored the extent to which a systematic review of audit and feedback could provide practical guidance about whether audit and feedback should be used to improve quality of diabetes care and, if so, how audit and feedback could be optimised. Based upon discussions with those responsible for conducting audit and feedback at a local level as well as our own experiences of doing so, we identified several questions which would be relevant to any attempt to operationalise audit and feedback in a healthcare service setting.

• Does audit and feedback work for this condition and setting, specifically improving the care of patients with a chronic disease – diabetes mellitus – in primary care?

• Does it work equally across all dimensions of care – from simple recording of cardiovascular risk factors to more complex areas of care such as glycaemic control? The latter requires a greater number of actions to achieve which include measuring blood glucose levels, reviewing the patient, checking compliance with drug and dietary therapies and checking patients' understanding of the condition.

• How should it be prepared? Should data be comparative and if so, what should the comparator group be? Should data be anonymised?

• How intensive should feedback be? Intuitively, providing more and personalised feedback on a recurrent and regular basis should have a greater impact on practice than a one-off report of (say) PCT-level aggregated data. However, it is uncertain whether the extra time and costs of ongoing data collection and preparing more frequent feedback would be matched by additional benefits.

• How should it be delivered – by post or by a messenger in person? And if by a messenger who should this be? Professionals might be more convinced by a message delivered by a colleague with a recognised interest in diabetes care rather than a non-clinical facilitator.

• What activities, if any, should accompany feedback? The likely costs and possible benefits of (say) educational meetings or outreach visits need to be weighed up against providing feedback via paper or computerised formats alone.

• What should be done about the poorest performers detected by the audit? Targeting such practices may help close the gap between the poorest and best performers. Alternatively, spreading effort to improve quality more equally amongst all practices may improve average performance for the whole PCT.

## Results

### The evidence from the systematic review

We identified a systematic review of audit and feedback that identified and appraised 85 randomised trial.[5] Audit and feedback was used for a wide range of clinical topics and problems. The review conclusions were:

• audit and feedback can improve professional practice, although the effects are generally small to moderate

• effectiveness varies substantially among different studies

• variation may be related to different methods of providing feedback or contextual factors, such as targeted behaviours and professionals

The review identified only five direct (head-to-head) comparisons of different methods of providing feedback (Table 4). One comparison suggested that feedback by a peer was more effective than that by a neutral observer [10]; another that feedback from a peer physician was no more effective than that from a nurse.[11] The other three comparisons found no effects related to recipients (group or individual) or content of feedback. None of these studies reported an economic evaluation.

**Table 4:** Evidence for questions addressed by the Cochrane Review.

**Questions**	**Most relevant analyses from Cochrane Review**	**Evidence from all trials reviewed (n = 85)**	**Evidence from chronic disease management trials (n = 15)**	**Evidence from trials of diabetes care (n = 4)**
**Does audit and feedback work?**	Any intervention involving audit and feedback versus no intervention +/- educational materials	83 comparisons: for dichotomous outcomes, median adjusted relative risk (RR) of non-compliance was 0.85 [Interquartile range (IQR) 0.74 to 0.96]*	Small to moderate effects in 11 of 19 comparisons	Moderate to large effects in two comparisons [12;13]
	
	Audit and feedback versus other interventions	Five comparisons: two show audit and feedback more effective than reminders; one that local opinion leaders more effective; one no effect over patient education; one no effect of audit and feedback with educational meetings over educational meetings alone	Small effect of audit and feedback over reminders from one comparison	None

**Does it work equally across all dimensions of care?**	No direct comparisons; exploration of heterogeneity	No heterogeneity explained by complexity of the targeted behaviour	None	None

**How should it be prepared? Should data be comparative and if so, what should the comparator group be? Should data be anonymised?**	*Content*. Patient information, such as blood pressure or test results, compliance with a standard or guideline, or peer comparison; versus information about costs or numbers of tests ordered or prescriptions	Two comparisons: no difference between peer comparison and individual feedback without peer comparison; nor between feedback on medication and feedback on performance	No difference between feedback on medication versus feedback on performance in one comparison	None

**How intensive should feedback be?**	*Recipients*. Individual or group	No difference between individual versus group feedback in one comparison	None	None
	
	*Frequency*. Once only or more frequent feedback	None	None	None
	
	*Length*. Once only feedback versus audit and feedback over a period of time	None	None	None
	
	Short term effects compared to longer term effects after audit and feedback stops	Mixed results from 11 comparisons	No difference from one comparison [14]	No difference from one comparison [14]
	
	Exploration of heterogeneity	No heterogeneity explained by intensity of audit and feedback		

**Questions**	**Most relevant analyses from Cochrane Review**	**Evidence from all trials reviewed (n = 85)**	**Evidence from chronic disease management trials (n = 15)**	**Evidence from trials of diabetes care (n = 4)**

**How should it be delivered – by post or by a messenger in person? And if by a messenger who should this be?**	*Format*. Verbal, written or both	None	None	None
	
	*Source*. Influential source [seen to be credible and trustworthy by the professional] or feedback from any other source	Two comparisons: peer feedback better than non-physician observer feedback; no difference between peer physician versus nurse feedback	No difference between peer physician versus nurse feedback in one comparison [11]	No difference between peer physician versus nurse feedback in one comparison [11]

**What activities, if any, should accompany feedback?**	Audit and feedback with complementary interventions versus audit and feedback alone	No clear effect of complementary interventions from 14 studies including various comparisons except for small effect of audit and feedback combined with educational outreach. Lower baseline compliance associated with larger effect sizes.	Small or mixed effects in two out of four comparisons	Outreach by peer or nurse more effective than feedback alone [11]

**What should be done about the poorest performers detected by the audit?**	None	None	None	None

*Relative risk [RR] is given for non-compliance. Therefore a lower RR is equivalent to greater effect size.

The review also evaluated 14 direct comparisons of audit and feedback alone compared to audit and feedback combined with other interventions (multifaceted interventions). There was no evidence that multifaceted interventions worked better than audit and feedback alone. A multivariate analysis explored potential causes of heterogeneity in the results (study quality, whether audit and feedback was combined with other interventions, intensity of feedback, complexity of the targeted behaviour, and level of baseline compliance). Only low baseline compliance was associated with greater effect sizes for multifaceted interventions. There was no evidence of larger effects with increasing intensity of feedback.

### The evidence for chronic disease management

Fifteen studies relate to chronic disease management (hypertension, diabetes, cholesterol control, depression, asthma and end-stage renal failure). Just over half of comparisons indicated that audit and feedback was more effective than doing nothing (Table 4). Using multifaceted interventions or modifying feedback methods did not enhance effectiveness.

### The evidence for diabetes care

Four studies evaluated audit and feedback in diabetes care, three set in primary care. Two comparisons addressed one of our key questions (Does audit and feedback work for this condition and setting?) and showed that audit and feedback, with or without other interventions, was more effective than doing nothing.[12,13] A UK primary care study.[12] showed that a multifaceted intervention incorporating low intensity audit and feedback moderately improved practice, specifically recording of key variables (e.g. glycaemic control, smoking habit). Audit and feedback also moderately increased US primary care physician compliance with guidelines.[13]

Two studies partially addressed three of our key questions about how to conduct audit and feedback (How intensive should feedback be? What activities, if any, should accompany feedback? How should feedback be delivered?). In US secondary care, there was no difference between continuing feedback against withdrawal of feedback in the accuracy of capillary blood glucose monitoring.[14] An Australian study of GPs found a small benefit of feedback given by a doctor or nurse compared with feedback alone, although it is difficult to judge whether the benefits of this approach outweighed the additional costs.[11] There was no difference in effect size between doctor and nurse feedback in this comparison. There was no relationship between study effect size and feedback intensity, co-intervention use or complexity of targeted behaviour across the four studies.

## Discussion

The review evidence was of limited use in informing the operationalisation of evidence based audit and feedback. A number of issues contributed to this – the heterogeneity of the studies in the overall review, the problems of interpreting sub-groups of studies within the larger review, and the lack of direct evidence (particularly from head-to-head comparisons) to answer key questions.

It is unclear how to use the review to extract generalisable lessons about how audit and feedback achieves its effects. For example, individual level feedback could reasonably be assumed to be more personally relevant and persuasive and thus more effective than feedback at a group level; there are no such direct comparisons available. Four out of 10 studies using individual feedback for chronic disease management reported no effect whilst both studies using group feedback reported positive effects. Therefore group feedback might be more effective than individual feedback, possibly by promoting peer pressure, consensus and subsequent action. Unfortunately this must all remain conjecture given the paucity of data to test different hypotheses about the causal mechanisms that make audit and feedback work.

Based upon a limited number of comparisons, audit and feedback appears to work better for diabetes than for other conditions. It is unclear whether this is because there is something intrinsically different about diabetes (or the audit methods used in diabetes) compared to other conditions, or whether this is an unreliable sub-group analysis of four studies selected from the 85 available. It is hard to have confidence in the findings of the diabetes studies in the absence of good, preferably *a priori*, reasons as to why these studies should be examined separately from others in the review.

Similar pitfalls exist in judging the relative effectiveness of different feedback methods. This is mainly because of the limited number of head-to-head comparisons comparing audit and feedback alone against combined interventions or variations in providing feedback. Across all studies, audit and feedback alone appears similarly effective to multifaceted strategies. However, the lack of difference in effect size could have occurred because multifaceted strategies were used in situations where investigators judged them necessary to overcome greater obstacles to improving care. In the absence of primary studies, the review cannot address some of the key questions such as whether intensive feedback would improve more complex outcomes (gylcaemic control) at an acceptable cost.

Mapped back onto the principles for good clinical audit, the evidence only supports doing audit if there is low baseline compliance (Table 3). This evidence relates to situations where there is low mean baseline compliance across all study physicians rather than relating solely to a selected “low compliance” group. Thus it is of no direct relevance to the key question of whether or not audit and feedback can promote change in poorly performing individuals. However, a baseline audit of multiple aspects of diabetes care would enable targeting of implementation activities at areas of low compliance.

The issues of external validity of randomised controlled trials (and by inference, systematic reviews) have been aired in the context of clinical studies.[15,16]. However, what we have had to deal with here is more to do with inadequate description of the interventions in the primary studies and an inadequate understanding of the causal mechanisms by which the intervention or its variants might exert their effects. Thus this lack of fundamental understanding accounts for the impossibility of assessing a behaviour change interventions' applicability to a particular service setting. We are a long way from being able to do what is now commonplace with clinical studies in terms of assessing the applicability of a clinical study to an individual patient.[17]

A rational approach to this situation is to develop a conceptual framework within which to describe common elements of settings, individuals, targeted behaviours and interventions [18-20]. This would enable the identification of features that systematically influence the effectiveness of interventions. For example, the effectiveness of audit and feedback may be influenced by factors such as health professionals' motivation to change or perceived peer pressure – generalisable concepts that can be used across different contexts. Behavioural theory can identify potentially modifiable factors underlying professional behaviour in order to identify those processes to target with an intervention. Hence, if perceived peer pressure was predictive of adherence to good practice criteria, feedback incorporating peer comparison might enhance effectiveness. This approach potentially offers a method for more effective selection and development of interventions to improve practice. The longer term possibility is to establish a theoretically grounded basis for selecting or tailoring interventions given specific barriers and circumstances. This would apply to all behaviour change strategies, not just audit and feedback.

## Conclusion

Review evidence was of limited use in informing the operationalisation of evidence based audit and feedback. This is mainly because of the heterogeneity of the studies in the overall review, the problems of interpreting sub-groups of studies within the larger review, and the lack of head-to-head comparisons to answer key questions. Audit and feedback will continue to be an unreliable approach to quality improvement until we learn how and when it works best. Conceptualising audit and feedback within a theoretical framework offers a way forward.

## Abbreviations

UK NHS United Kingdom National Health Service

NSF National Service Framework

GP General Practitioner

NICE National Institute for Clinical Excellence

HbA1c Glycosylated haemoglobin

PCT Primary Care Trust

## Competing interests

The author(s) declare that they have no competing interests.

## Authors' contributions

GJ and JY undertook the Cochrane Review of audit and feedback. RB co-authored *Principles for Best Practice in Clinical Audit. *ME suggested the idea for this paper. RF wrote the first draft and is guarantor. All other authors helped draft the manuscript and have approved the final manuscript.

## Pre-publication history

The pre-publication history for this paper can be accessed here:


